# Large pancreatic acinar cell carcinoma mass excision

**DOI:** 10.1093/jscr/rjag443

**Published:** 2026-06-10

**Authors:** Ari Solomon, Laura Roberson, Karim Jreije

**Affiliations:** Community Memorial Hospital System, 147 N Brent Street, Ventura, CA 93003, United States; Department of Trauma, Ventura County Medical Center, 300 Hillmont Ave, Ventura, CA, 93003, United States; Department of Trauma, Ventura County Medical Center, 300 Hillmont Ave, Ventura, CA, 93003, United States

**Keywords:** acinar cell carcinoma, pancreatic mass, hepatobiliary mass, splenectomy

## Abstract

Pancreatic acinar cell carcinomas are rare malignant exocrine neoplasms. Due to their rarity, current treatments are being researched. Most tumors range from 5 to 10 cm in size. This case report describes a 15 cm tumor that was resected en bloc with no local recurrence at 2 months.

## Introduction

Pancreatic acinar cell carcinoma (PACC) is a rare malignant neoplasms, accounting for approximately 1%–2% of all pancreatic cancers, ranging from 5 to 10 cm in size. Due to their rarity, PACC remains poorly understood, and much of the current knowledge and management are derived from case series and individual case reports. This case report describes a very large (15 cm) PACC, the clinical presentation, diagnostic workup, surgical management, pathological features, and clinical course thereby contributing to a better understanding of this uncommon pancreatic malignancy.

## Case report

**Figure 1:**
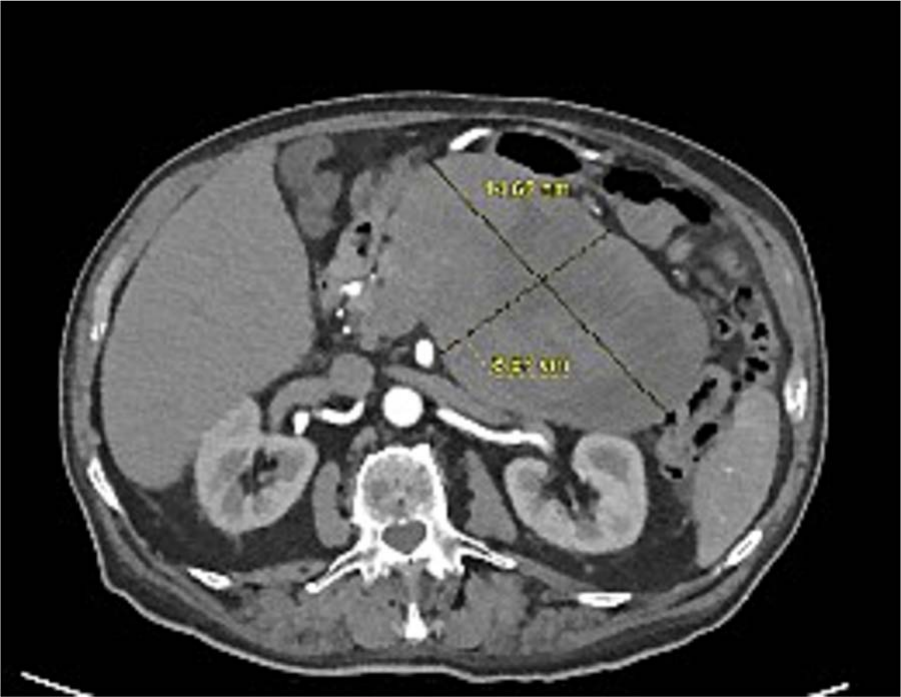
Transverse high resolution CT of the PACC prior to surgery.

**Figure 2:**
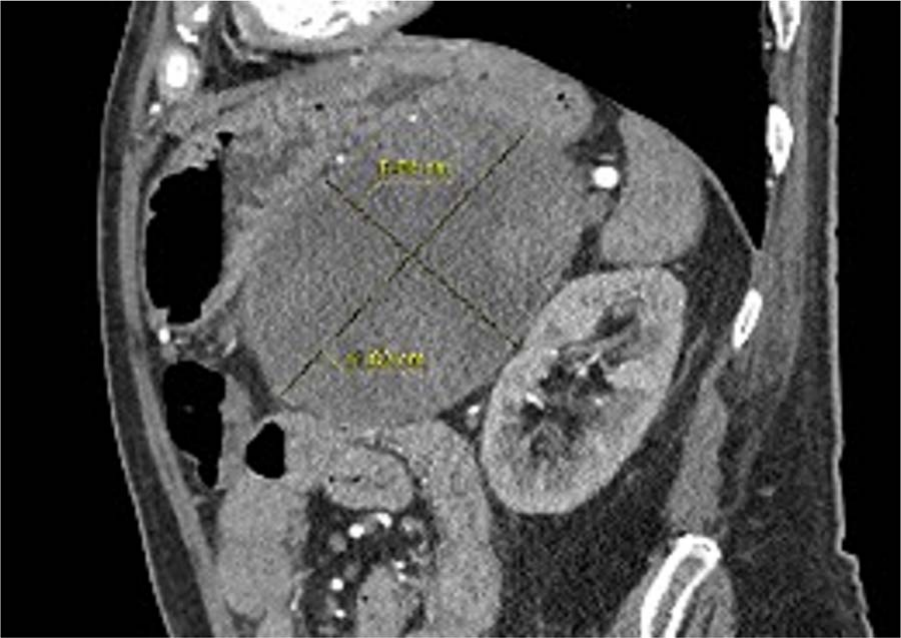
Sagittal high resolution CT of the PACC prior to surgery.

An 82-year-old male with the medical history of hypertension, diabetes, hypothyroidism, and invasive metastatic melanoma of the cheek underwent a screening positron emission tomography/computed tomography (PET/CT) and a large 11 cm mass abutting the stomach was discovered (Figs 1 and 2). He then underwent endoscopic ultrasound and biopsy which demonstrated acinar cell carcinoma. High resolution CT was then obtained which showed a large 15 cm heterogeneous mass occupying the pancreatic body and tail abutting the stomach, without any pancreatic duct dilation. Surgical planning was conducted and he ultimately underwent an open distal pancreatectomy and splenectomy with enbloc resection of the mass. A laparoscopic approach was initiated; however, the decision to convert to an open procedure was made after evaluating the size laparoscopically. During his hospitalization, he initially had high drain output (amylase negative) which eventually improved. He ultimately discharged home on post op day 4. His drain was eventually removed during a post-operative visit. He continued post-operative follow-up with oncology and remains disease free to date of this publication.

Surgical pathology resulted as a 17.5 × 10 × 8.5 cm PACC with negative lymph nodes (0/3) and negative margins. The specimen stained positive for CK7, CAM5.2, BCL10, Chymotrypsin, Trypsin, and had a Ki-67 of 25%, consistent with PACC. The patient’s TNM staged was classified as pT3N0. The spleen had no significant histopathologic abnormalities.

**Figure 3:**
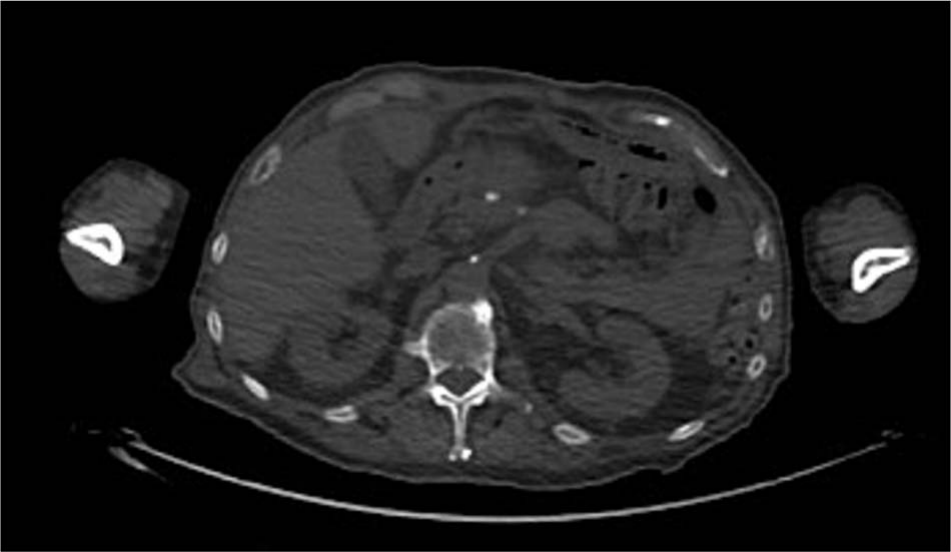
Two month surveillance CT demonstrating complete resection without recurrence.

There were no signs of local recurrence on surveillance imaging at 2 months; however, the PET did show a 2 cm submandibular lymph node that was later biopsied and found to show recurrent metastatic melanoma with high SOX10 mutation (Fig. 3). He is currently scheduled to undergo further resection for the melanoma and will have additional surveillance for the PACC.

## Discussion

PACC is relatively rare and arise from the exocrine acinar cells of the pancreas, which produce digestive enzymes [1]. They are much less common than other forms of pancreatic cancer such as ductal carcinomas but tend to yield a better prognosis [2]. They clinically present with nonspecific symptoms such as abdominal pain, bloating, and or nausea, but are sometimes incidentally discovered as in this case report. In some cases, patients may develop excessive enzyme production related symptoms including subcutaneous fat necrosis and polyarthralgia [3]. However, only a small percentage of patients with PACC presents with the classically taught association of Schmid’s triad: panniculitis, polyarthritis, and eosinophilia. Radiographically, PACC appears as large, well-circumscribed masses that can look similar to other pancreatic tumors. On average, PACC is 5–10 cm at the time of diagnosis [4]. Molecularly, PACC has fewer KRAS mutations but higher alterations of APC proportionally [5].

They are more commonly found in males and have a bimodal distribution as seen in both kids and adults [1]. Current practice guidelines suggest surgical resection with negative margins as the first line treatment although there is no consensus. Even with local metastasis, current guidelines suggest surgical management while adjuvant chemotherapies and genetic testing for PACC are currently being researched [6]. There have been several case studies that suggest surgical resection has decreased local recurrence but research is currently investigating the use of neoadjuvant and adjuvant chemotherapies along with targeted genetic therapies [7]. One of the larger comparative case–control studies found that the liver was the most common site of metastasis with lymph nodes involvement found in over 40% of all unresectable tumors. Additionally, the majority of resectable tumors included enbloc resection of surrounding organs (spleen, duodenum). Lymphovascular and perineural invasion were found to be the strongest prognostic factors [7].

This case study reports one of the largest known PACCs measuring 17.5 cm in the greatest dimension. Given the lack of published data and guidelines, we employed surgical resection as the primary modality after a multidisciplinary preoperative workup. Complete resection with negative margins with no local metastasis at the time of operation yielded disease free follow-up in this case without adjuvant therapies. This case highlights the importance of utilizing a multidisciplinary approach to management of PACC. The lack of robust guidelines and standards also highlights the importance of publishing PACC cases. Post-surgical management also lacks uniformity given the rarity of these cases and additional research and case studies can help develop more optimal management.

## Conclusion

PACC is a rare tumor. Currently, there are no best practice/management guidelines in treatment given the rarity of these tumors. This case highlights the largest published PACC to date. We employed an enbloc surgical resection approach to management and at the time of publication, lack recurrence without adjuvant treatments although will need to continue with postoperative surveillance to better assess clinical response long term.
